# Difficult Behaviors in the Emergency Department: A Cohort Study of Housed, Homeless and Alcohol Dependent Individuals

**DOI:** 10.1371/journal.pone.0124528

**Published:** 2015-04-28

**Authors:** Tomislav Svoboda

**Affiliations:** Department of Family and Community Medicine, University of Toronto, Centre for Research on Inner-City Health, Li Ka Shing Knowledge Institute—St. Michael’s Hospital, Toronto, Canada; University of California San Diego, UNITED STATES

## Abstract

**Background:**

This study contrasted annual rates of difficult behaviours in emergency departments among cohorts of individuals who were homeless and low-income housed and examined predictors of these events.

**Methods:**

Interviews in 1999 with men who were chronically homeless with drinking problems (CHDP) (n = 50), men from the general homeless population (GH) (n = 61), and men residing in low-income housing (LIH) (n = 58) were linked to catchment area emergency department records (n = 2817) from 1994 to 1999. Interview and hospital data were linked to measures of difficult behaviours.

**Results:**

Among the CHDP group, annual rates of visits with difficult behaviours were 5.46; this was 13.4 (95% CI 10.3–16.5) and 14.3 (95% CI 11.2–17.3) times higher than the GH and LIH groups. Difficult behaviour incidents included physical violence, verbal abuse, uncooperativeness, drug seeking, difficult histories and security involvement. Difficult behaviours made up 57.54% (95% CI 55.43–59.65%), 24% (95% CI 19–29%), and 20% (95% CI 16–24%) of CHDP, GH and LIH visits. Among GH and LIH groups, 87% to 95% were never involved in verbal abuse or violence. Intoxication increased all difficult behaviours while decreasing drug seeking and leaving without being seen. Verbal abuse and violence were less likely among those housed, with odds ratios of 0.24 (0.08, 0.72) and 0.32 (0.15, 0.69), respectively.

**Conclusions:**

Violence and difficult behaviours are much higher among chronically homeless men with drinking problems than general homeless and low-income housed populations. They are concentrated among subgroups of individuals. Intoxication is the strongest predictor of difficult behaviour incidents.

## Introduction

Individuals who are homeless use emergency departments more heavily than the general population, frequently accessing them as sources of primary care.[1–6] Barriers to accessing care in such settings has been described in the general homeless population and the role of difficult behaviours or interactions with staff has been raised.[7,8] Currently the only available data about homelessness and difficult behaviours shows that homelessness and alcohol use are risk factors.[9,10] Individuals who are chronically homeless and heavy alcohol users form a recognizable subset of the homeless population and are anecdotally known to experience more difficulties with behaviours.[11–15] Terms such as “public inebriates,” “chronic inebriates,” and “frequent fliers” have been used in the medical and lay literature and may reflect a level of visible importance for this subgroup that has led to stigmatization rather than understanding.[16,17] Despite their visibility, policy relevance and need for understanding, information is generally anecdotal with few epidemiological studies of this group. Misunderstandings or misplaced opinions of difficult behaviours in these populations may serve to further stigmatize and marginalize rather than help these already vulnerable groups. Difficult behaviours generally refer to physical or verbal abuse or violence that might discourage staff from providing care due to the threat these behaviours may pose.[18–21] Absent or less well described are non-threatening behaviours that still pose significant barriers to complete assessments, treatments or proper discharge and follow-up plans. These might include difficult histories or leaving before being seen. This retrospective cohort analysis of emergency department records was carried out to answer the following clinically and policy relevant questions: 1) what difficult behaviours are reported by care providers? 2) what proportion of individuals engage in difficult behaviours? 3) how frequently? and 4) what are the risk factors and distribution of different difficult behaviours? These questions were explored in three policy relevant groups: those who are chronically homeless and heavy users of alcohol, those who are in the general homeless population and those who live in low-income housing.

## Methods

### Design

This study analyzed data from a larger study evaluating the impact of a harm reduction program on individuals who were chronically homeless with severe drinking problems (CHDP) using individuals within low income housing (LIH) and general homeless (GH) populations as non-equivalent controls.[22] A retrospective cohort analysis was used. This approach is useful when resource constraints and characteristics of a study population make it difficult to follow cohorts over time. For this analysis, rates of difficult behaviours found in emergency department records during a six year period prior to subject interviews were compared among the three groups of men.

### Ethics Statement

Independent research ethics review and approval of the larger study was obtained from ethics review committees in the University of Toronto Office of Research Services, St. Michael's Hospital, the University Health Network, and St. Joseph’s Health Centre. Written informed consent was obtained from all participating study subjects.

### Setting and Participants

All adult men were invited to participate from a 60 bed shelter-based alcohol harm reduction program (CHDP) designed for chronically homeless men unable to abstain from alcohol and rejected from typical shelters due to their drinking patterns; a 250 bed men’s shelter for the general homeless population (GH) and three low income housing sites (LIH). The subjects were recruited in April and May 1999. Data from hospital records were abstracted for the period January 1, 1994, to April 14, 1999 inclusive. The two control group samples were identified using systematic sampling with random start, a type of sampling which approximates a simple random sample. Every third individual entering the participating sites during blocks of time spanning the whole time that individuals enter the sites were invited to meet with an interviewer. Harm reduction program recruits not meeting alcohol dependence criteria were excluded from the analysis (n = 9) as they represented residents sheltered for problems unrelated to alcohol use.

### Free and informed consent for vulnerable subjects

Recruitment methods to obtain free and informed consent for study participation included recruiter discussion, written information on the consent form, researcher availability to answer questions any time during the study, recruiter training, and competence assessments where recruiters asked subjects to express in their own words 1) the purpose of the study, 2) that only researchers would access their records and 3) how they could withdraw from the study at any time. Participation was hidden from agency staff and express statements were made to clients that their care would not be disadvantaged by non-participation. Additional techniques shown to improve comprehension included: using a lower reading level [23], using larger typeface [24], quizzing [25] and having multiple individuals provide the information.[26]

### Data sources and variables

Subject records in all 9 downtown Toronto hospitals were reviewed and validated and study-specific instruments determined subjects’ alcohol dependence status, history of homelessness, and length of time residing in the study hospitals’ catchment area. Data from physician, nurse, and paramedic elements of emergency department records were abstracted using computer forms and pre-defined coding lists and definitions. Abstracted data included: 1) intoxication; and head injury 2) behaviours including: violent behaviour (aggressive, threatening, abusive, etc.); verbal abuse; uncooperative; drug seeking; difficult historian; leaving against medical advice; leaving without being seen by physician; and leaving before discharge and 3) interventions including: presence or involvement of security staff during medical encounter; escort out by security staff; and use of restraints. Behaviours were rated as severe if they included related qualifiers (e.g. “very,” “++,” etc.); a raised voice or threats to staff; or if security or restraints were involved.

The rates of emergency department visits were determined using the total group observation time. Subjects with observation times greater than 6 months were used to determine what proportion of subjects accounted for the majority of annual difficult incidents that occurred for each group.

### Reducing Sources of Bias

CHDP clients were approached up to three separate times to reduce the chances of non-selection among more severely intoxicated clients. To maximize response rate in all groups we provided a $20 honorarium for completed interviews, and utilized staff familiar and trusted by clients to introduce recruitment staff using uniform scripts. Standardized training, scripts and probes, supervision by the principal investigator and blinding to group membership reduced interviewer measurement biases. To reduce hospital chart abstraction biases and increase reliability, the abstractors were blind to study group membership, and each record was reviewed twice by two independent teams. To reduce missing hospital records with poorly recorded identifiers due to intoxicated presentations, records were hand-searched by two independent study staff, then further probabilistically matched to the interview subjects. Multiple identifiers were collected including health insurance numbers, and alias names. Data from the time subjects reported living outside the hospital catchment areas, were excluded. Power calculations for detecting changes in social service use in the original study determined sample sizes.

### Analyses

Confidence intervals for proportions of visits involving difficult behaviors used a normal approximation of the binomial distribution.[27] Variables predicting the occurrence of difficult behaviours were determined using multivariate logistic regression, controlling for individual subjects and using a binary distribution for the outcome (SAS 9.0 Glimmix Procedure, 2010).

## Results

Of the 227 individuals approached for the study, 178 (78%) agreed to participate. In the CHDP group, 9 did not meet alcohol dependence criteria and were excluded from further analysis. Table 1 compares demographic, mental health measures and emergency department visit rates among the three groups. The CHDP group tended to be older, homeless longer and have more emergency department visits than the other groups.

**Table 1:** Comparison of Demographic and Mental Health Measures between study subject cohorts.

Characteristic	Chronically homeless with drinking problems (CHDP) n = 50(95% CI)	General Homeless (GH) n = (95% CI)	Low-Income Housed (LIH) Cohort n = (95% CI)
Median Age	45–49	30–34	45–49
single	46% (33%-60%)	72% (61%-86%)	68% (56%-82%)
post-secondary education^†^	22% (12%-34%)	26% (15%-38%)	24% (13%-35%)
Life time years homeless^†^	13.9 (11.2,16.7)	5.0 (3.3, 6.6)	2.5 (1.5, 3.5)
Years of residence in inner city study area^†^	17.8 (13.9, 21.8)	5.5 (3.7,7.2)	13.1 (8.9,17.2)
Major Depressive Episode in past year^†^ ^‡^	6.9% (0.3%-13.6%)	9.0% (1.6%-16.6%)	5.6% (0%-11.7%)
Generalized Anxiety Disorder in past year^†^ ^‡^	12.1% (3.7%-20.8%)	10.2% (2.4%-18.4%)	18.9% (8.8%-29.6%)
Drug Dependence in past year^†^	22.4% (11.7%-33.9%)	11% (2.8%-20%)	5.1% (0.0%-10.8%)
Alcohol Dependence in past year^†^	100%	30% (18%-43%)	23% (12%-34%)
Emergency Department visits in past year	9.5 (9.09–9.90)	1.54 (1.36–1.72)	1.81 (1.64–1.98)

^*^ CI denotes confidence interval

^†^ After direct age standardized to the CHDP cohort.

^‡^Alcohol dependence, Major Depressive Episode, Generalized Anxiety Disorder and Drug Dependence meeting DSM IIIR criteria using the WHO Composite International Diagnostic Interview Short Form instrumen

The study spanned 641 subject years and 2817 emergency department records were reviewed. The absolute annual crude rate of emergency department visits with any difficult behaviour was 5.46 (95% CI 5.16–5.77) for the CHDP group; this was 13.4 (95% CI 10.3–16.5) times higher than the GH group with a rate of 0.408 (95% CI 0.315–0.500) and 14.3 (95% CI 11.2–17.3) times higher than the LIH group with a rate of 0.383 (95% CI 0.304–0.462). More than half (0.575, 95% CI 0.554–0.597) of the visits by the CHDP group included difficult behaviours. This was more than double the proportion for the GH group (0.24, 95% CI 0.19–0.29) and the LIH group (0.20, 95% CI 0.16–0.24), who were not significantly different (p = 0.18).

Table 2 shows a similar proportional breakdown of difficult behaviours among the three groups. There were more verbal abuse incidents and security staff interventions in the CHDP group. Uncooperative behaviours within the medical encounter were higher in the CHDP group. In the groups combined, severe difficult behaviours were 18.4% (95% CI 16.4–20.4%) of all difficult behaviours, and severe violent behaviours were 43.2% (95% CI 36.8–49.5%) of all violent behaviours; the three groups were not significantly different from each other.

**Table 2:** Breakdown of types of difficult behaviour during emergency department visits among homeless and low-income housed men.

Variable	All Groups		Chronically Homeless with Drinking Problems (CHDP) Group		General Homeless (GH) Group		Low-Income Housed (LIH) Group	
	N	Proportion of visits as percent	N	Proportion of visits as percent	N	Proportion of visits as percent	N	Proportion of visits as percent
Any difficult behaviour	1378	100	1213	100	75	100	90	100
Violent behaviour	251	18.2 (16.2, 20.3)	234	19.3 (17.1, 21.5)	8	10 (4, 20)	9	10 (4, 20)
Verbal abuse	346	25.1 (22.8, 27.4)	331	27.3 (24.8, 29.8)	9	10 (5, 20)	6	7 (2, 10)
Uncooperative	1022	74.17 (71.85, 76.48)	902	74.4 (71.9, 76.8)	52	69 (59, 80)	68	76 (67, 84)
Drug seeking	248	18 (16, 20)	233	19.2 (17.0, 21.4)	9	10 (5, 20)	6	7 (2, 10)
Difficult historian	125	9.1 (7.6, 10.6)	104	8.60 (7.00, 10.1)	6	8 (2, 10)	15	17 (9, 24)
Security involved	152	11.0 (9.38, 12.7)	143	11.8 (10.0, 13.6)	6	8 (2, 10)	3	3 (-4, 7)
Violent behaviour	251	100	234	100	8	100	9	100
Involving security	31	12 (10, 15)	30	13 (8, 17)	1	10 (10, 20)		
Use of restraints	107	42.6 (36.5, 48.7)	97	41 (35, 48)	7	90 (60, 110)	3	30 (3, 60)
Physical assaults	5	2 (0.3, 4)	5	1 (0.3, 4)				
Use of restraints	107	100	97	100	7	100	3	100
Patient intoxicated	94	88 (82, 94)	88	91 (85, 96)	4	60 (20, 90)	2	70 (10, 120)
GCS < 9	26	24 (16, 32)	24	25 (16, 33)	1	10 (-10, 40)	1	30 (-20, 90)
All uncooperative behaviours	1022	100	902	100	52	100	68	100
Uncooperative in encounter	490	47.9 (44.9, 51)	461	51.1 (47.8, 54.4)	12	23 (12, 35)	17	25 (15, 35)
Left against medical advice	109	10.7 (8.8, 12.6)	94	10 (8.4, 12)	7	10 (4, 20)	8	10 (4, 20)
Left without being seen	413	40.4 (37.4, 43.4)	353	39.1 (36, 42.3)	26	50 (36, 64)	34	50 (38, 62)
Left before discharge	230	22.5 (19.9, 25.1)	195	21.6 (18.9, 24.3)	16	31 (18, 43)	19	28 (17, 39)
All security involvement	152	100	143	100	6	100	3	100
Security at bedside	66	43 (36, 51)	59	41 (33, 49)	5	80 (50, 100)	2	70 (10, 100)
Security involved due to uncooperativeness	8	10 (2, 10)	8	6 (2, 9)				
Security escort out of department	55	36 (29, 44)	54	38 (30, 46)			1	30 (-20, 90)

All bracketed figures are 95% confidence intervals.

Table 3 summarizes what proportion of subjects accounted for most of the difficult behaviour incidents. In all types of incidents the proportion was much higher in the CHDP group. The median proportion of visits with any difficult behaviour among subjects in the CHDP group was 0.50 (IQR 0.35–0.59). This was significantly higher than for the GH and LIH groups, which had medians of 0.00 (IQR 0.0–0.23) and 0.00 (IQR 0.0–0.33), respectively.

**Table 3:** Proportion of subjects who account for annual aggressive and difficult behaviour incidents in each study group.

	Proportion Who Account for Ninety Percent of Incidents				Proportion Who Account for All Incidents			
	All Groups Percent	CHDP Group Percent	GH Group Percent	LIH Group Percent	All Groups Percent	CHDP Group Percent	GH Group Percent	LIH Group Percent
Any difficult behaviour	31.79	51.06	20.83	25	55.63	95.74	35.42	39.3
Violent behaviour	17.9	36.2	6.25	7.1	25.8	63.8	8.33	8.9
Verbal abuse	15.2	36.2	10	5	30.5	78.7	10	5
Uncooperative	31.79	53.2	19	25	53	95.7	31	36
Drug seeking	9.3	12.8	4	4	15	38.3	6	4
Difficult historian	25.2	48.9	4	5.4	27.8	74.5	4	8.9
Security involvement	15.9	27.7	6	4	19.9	48.9	8	5
Violent behaviour	17.9	36.2	6.25	7.1				
Involving security	9.9	23	2	0	9.9	30	2	0
Use of restraints	16.6	38	4	4	20.5	55	6	4
Physical assault	1	4	0	0	1	4	0	0
Use of restraints	16.6	38	4	4	20.5	55	6	4
Patient intoxicated	15	34	4	2	18	49	6	2
GCS < 9	9.3	21	2	2	9.9	28	2	2
All uncooperative behaviours	31.79	53.2	19	25				
Uncooperative in encounter	24.5	53.2	13	13	40.4	93.6	17	16
Left against medical advice	19.2	38	8	10	25.8	57	10	10
Left without being seen	27.2	44.7	8.3	14	35.1	78.7	13	18
Left before discharge	25.8	46.8	17	13	35.1	70.2	23	16
All security involvement	15.9	27.7	6	4				
Security at bedside	13	23	6	4	15	38	6	4
Security involved due to uncooperativeness	4	10	0	0	5	10	0	0
Security escort out of department	7.9	13	0	2	7.9	23	0	2

Table 4 shows the proportion of subjects who fell within each of four different ranges of difficult behaviour rates. For example, 38% of those in the CHDP group were noted to be drug seeking, with 2% drug seeking more than half of the time, while in the GH and LIH groups only 5% of subjects were ever involved in drug-seeking behaviours and these occurred in less than 50% of their visits.

**Table 4:** Proportion (%) of subjects with varying rates of difficult behaviours recorded in emergency department records during emergency room visits.

Variable	CHDP Group %				GH Group %				LIH Group %			
	0%	0.1–10%	0.1–50%	50.1–100%	0%	0.1–10%	0.1–50%	50.1–100%	0%	0.1–10%	0.1–50%	50.1–100%
Any difficult behaviour	6	0	50	44	67	3	26	7	58	5	32	10
Violent behaviour	38	34	62	0	90	5	10	0	90	3	10	0
Verbal abuse	24	32	76	0	87	8	10	0	95	3	5	0
Uncooperative	6	4	74	20	74	3	23	3	63	2	32	5
Drug seeking	62	30	36	2	95	2	5	0	95	2	5	0
Difficult historian	30	58	70	0	93	2	5	2	90	2	10	0
Security involvement	52	36	48	0	93	5	7	0	95	3	5	0
Violent behaviour	38	34	62	0	90	5	10	0	90	3	10	0
Involving security	72	24	28	0	98	2	2	0	100	0	0	0
Use of restraints	48	38	52	0	92	5	8	0	95	2	5	0
Physical assault	96	4	4	0	100	0	0	0	100	0	0	0
Use of restraints	48	38	52	0	92	5	8	0	95	2	5	0
Patient Intoxicated	54	34	46	0	95	3	5	0	97	2	3	0
Unconscious	74	24	26	0	98	2	2	0	98	0	2	0
All uncooperative behaviours	6	4	74	20	74	3	23	3	63	2	32	5
Uncooperative in encounter	10	20	84	6	85	10	10	0	83	2	20	2
Left against medical advice	44	44	56	0	92	5	8	0	88	5	10	0
Left without being seen	24	20	74	2	87	3	10	0	81	0	17	2
Left before discharge	32	38	68	0	82	5	18	0	85	5	20	0
All security involvement	52	36	48	0	93	5	7	0	95	3	5	0
Security at bedside	76	24	24	0	100	0	0	0	98	0	2	0
Security involved due to uncooperativeness	64	36	36	0	95	3	5	0	97	3	3	0
Security escort out of department	86	10	10	0	100	0	0	0	100	0	0	0

In Table 5, the multivariate modeling of all groups combined shows that difficult behaviours were significantly associated with intoxication. Subjects were intoxicated with alcohol alone 85.8% (95% CI 84.0–87.7%) of the time while the remaining intoxication involved other substances (9.5%, 95% CI 8.0–11%). Types of intoxication were similar across groups. Leaving without being seen and drug seeking were the only difficult behaviours that were less likely among those who were intoxicated; the remaining difficult behaviours were more likely among those intoxicated. The proportion of difficult behaviours in which intoxication was implicated was significantly greater in the CHDP group for verbal abuse (77%, 95% CI 72–81%, p = 0.01), physical violence (81%, 95% CI 75–85, p = 0.02), and leaving without being seen (49%, 95% CI 43–54%, p = 0.001).

**Table 5:** Odds ratios of factors impacts on rates of difficult behaviours in emergency departments based on a multivariate model that included all of these factors.

Difficult behaviour or incident	Intoxication during visit	Record of head injury	Major reduced level unconscious	Problem drinker	Age	Housed	Mood disorder at study follow up
Any difficult behaviour	1.79 (1.47, 2.17)*	0.96 (0.73, 1.26)	1.81 (1.24, 2.65)*	1.46 (0.91, 2.36)	1.01 (0.99, 1.03)	0.61 (0.36, 1.03)	0.86 (0.53, 1.39)
Violent behaviour	3.23 (2.29, 4.58)*	0.85 (0.55, 1.32)	3.97 (2.52, 6.26)*	1.03 (0.49, 2.16)	0.98 (0.96, 1.01)	0.50 (0.20, 1.26)	0.60 (0.32, 1.15)
Verbal abuse	2.91 (2.16, 3.92)*	1.13 (0.77, 1.67)	1.79 (1.12, 2.87)*	1.64 (0.76, 3.52)	0.99 (0.97, 1.01)	0.24 (0.08, 0.72)*	0.47 (0.24, 0.91)*
Uncooperative	1.71 (1.42, 2.07)*	1.09 (0.83, 1.43)	1.36 (0.95, 1.96)	1.38 (0.87, 2.19)	1.01 (0.99, 1.03)	0.65 (0.38, 1.09)	0.90 (0.57, 1.42)
Drug seeking	0.52 (0.35, 0.76)*	0.31 (0.12, 0.81)*	0.29 (0.07, 1.29)	3.45 (0.92, 12.90)	0.99 (0.95, 1.04)	0.82 (0.20, 3.47)	0.87 (0.28, 2.67)
Difficult historian	2.78 (1.76, 4.40)*	1.23 (0.74, 2.06)	0.85 (0.39, 1.84)	0.80 (0.38, 1.71)	1.00 (0.98, 1.02)	0.75 (0.32, 1.76)	1.32 (0.72, 2.41)
Security involvement	3.12 (2.04, 4.78)	0.93 (0.51, 1.70)	1.33 (0.66, 2.68)	1.15 (0.43, 3.08)	0.97 (0.93, 1.00)	1.01 (0.97, 1.04)	1.04 (0.47, 2.29)
Violent behaviour	3.21 (2.27, 4.54)	0.82 (0.52, 1.28)	4.09 (2.59, 6.47)	1.23 (0.61, 2.49)	0.98 (0.96, 1.01)	1.01 (0.98, 1.04)	0.60 (0.31, 1.19)
Involving security	Did not converge						
Use of restraints	5.16 (2.80, 9.52)*	0.60 (0.31, 1.16)	6.40 (3.67, 11.18)	0.56 (0.21, 1.52)	0.99 (0.96, 1.02)	0.28 (0.07, 1.16)	0.83 (0.36, 1.92)
Physical assault	Did not converge						
Use of restraints	5.08 (2.74, 9.41)	0.55 (0.28, 1.10)	6.84 (3.89, 12.03)	0.53 (0.19, 1.47)	0.99 (0.96, 1.02)	1.01 (0.97, 1.05)	0.93 (0.38, 2.26)
Patient intoxicated	Did not converge						
Unconscious	Did not converge						
All uncooperative behaviours	1.71 (1.42, 2.07)*	1.09 (0.83, 1.43)	1.36 (0.95, 1.96)	1.38 (0.87, 2.19)	1.01 (0.99, 1.03)	0.65 (0.38, 1.09)	0.90 (0.57, 1.42)
Uncooperative in encounter	4.08 (3.10, 5.37)*	1.46 (1.06, 2.00)*	2.25 (1.50, 3.37)*	0.90 (0.49, 1.65)	1.01 (0.99, 1.03)	0.32 (0.15, 0.69)*	0.72 (0.40, 1.27)
Left against medical advice	1.60 (1.01, 2.54)*	1.64 (0.98, 2.75)	2.13 (1.13, 3.99)*	1.15 (0.53, 2.51)	1.00 (0.98, 1.02)	0.68 (0.27, 1.71)	1.12 (0.62, 2.03)
Left without being seen	0.59 (0.47, 0.75)*	0.75 (0.49, 1.14)	0.21 (0.09, 0.50)*	2.40 (1.31, 4.41)*	1.00 (0.98, 1.02)	1.07 (0.55, 2.07)	0.97 (0.55, 1.72)
Left before discharge	1.79 (1.31, 2.46)*	0.82 (0.52, 1.30)	1.84 (1.12, 3.03)*	1.14 (0.67, 1.94)	1.00 (0.99, 1.02)	0.72 (0.39, 1.33)	1.13 (0.76, 1.66)
All security involvement	3.12 (2.04, 4.77)*	0.92 (0.50, 1.67)	1.33 (0.66, 2.67)	1.14 (0.43, 3.04)	0.97 (0.94, 1.00)*	0.34 (0.09, 1.35)	1.01 (0.48, 2.14)
Security escort out of department	1.84 (0.99, 3.42)	1.24 (0.50, 3.07)	0.48 (0.11, 2.07)	2.43 (0.47, 12.52)	0.94 (0.90, 0.99)*	0.56 (0.06, 5.02)	0.14 (0.03, 0.67)*
Security observation	3.91 (2.04, 7.48)*	0.39 (0.12, 1.30)	1.40 (0.52, 3.79)	1.28 (0.32, 5.16)	0.98 (0.94, 1.01)	0.50 (0.09, 2.74)	1.42 (0.59, 3.43)
Security involved due to uncooperativeness	Did not converge						

Table 6 provides difficult behaviour descriptions found in the emergency records.

**Table 6:** Sample difficult behaviour descriptions abstracted from emergency department records.

	Sample Behaviour Descriptions	More Severe Behaviour Descriptions
Violent behaviour	“slightly combative”	“restrained for own protection”
	“aggressive, violent”	“pt. attempting to punch crew!”
	“showed his ‘fists of steel’”	“verbally/physically abusive +++”
	“aggressive behaviour”	“patient put in 4 point restraints for being aggressive—security called to observe”
	“threatening behavior”	“patient very combative”
Verbal abuse	“verbally abusive to all staff encountered”	“threatened nurses, removed by security w/o being seen”
	“patient told he will not be given any Valium, became abusive and left”	“patient yelling at secretary”
	“patient stated: ‘f—k off’”	“discharged from hospital bc. he was harassing other patients”
	“singing obscene jingles”	“death threats to staff”
Uncooperative	“patient uncooperative”	“emptied bladder on floor”
	“patient pulled out IV”	“flopping around on ER floor security not there, escorted out by doctor”
	“removed own condom catheter and then voided in bed”	“threatening staff, peed on floor, locked self in room—escorted out by security”
	“refused treatment”	“patient ripped off all stabilization equip collar and straps screaming wants to be seen”
Uncooperative with care or encounter	“drinking cooking wine in ER”	“very uncooperative”
	“refusing BP”	“patient escorted out by security b/c he refused to leave ER”
	“advised to return meds since obviously non-compliant”	“restrained because patient ‘uncooperative’”
	“pt. is not actually suicidal but is seeking bed in ER”	“patient won’t sit down, won’t hand over glue, wants to leave, escorted out eventually by security”
Security observation		“security called to observe”
		“patient left then bought back by security”
		“escorted to waiting room by security”
Security involved due to uncooperativeness		“was caught drinking in the ER and escorted out by security”
		“asked by security to give up bottle of liquor”
		“antagonistic w/ security staff”
Security escort out of department		“escorted out by security”
		“removed by security w/o being seen”
		“patient abusive removed by security”
		“set bedsheets on fire, escorted out by police”
Physical assault involving security		“patient very violent, restrained + masked because patient spitting; screaming, belligerent”
		“patient extremely violent, spat in nurse face, removed by security”
		“became abusive w/staff, hit nurse”
Use of restraints		“very combative, 4pt restrain, threatening to kill everyone”
		“hospital asks to have him sent back by police, put in 4–pt restraint for staff safety”
		“put in 4–pt restraint for staff safety”
		“placed in 2–point restraint”
		“patient had to be put into 2patient restraint to be assessed”
		“patient brought in for chemical and physical restraint for attacking transit driver”
Restraints when unconscious		“intubated, sent to ICU, 2 point restraint because tried d/c ET tube”
		“patient collapsed”, “held patient down manually while IV established”
		“GCS = 9, placed in 4 point restraints”
Drug seeking	“frequent flier—drug seeker, wants Valium—as family doctor can’t prescribe”	
	“patient drug seeking”	
	“patient seeking prescription for Valium”	
Difficult historian	“difficult to obtain history”	
	“difficult historian”	
	“information unreliable and difficult to obtain”	
Left against medical advice	“patient left ‘AMA’”	
	“refused further assessment and walked out AMA”	
	“nurse started IV, patient pulled out IV and left AMA”	
	“left AMA, signed form”	
Left without being seen	“LWBS”	
	“was not seen by doctor”	
Left before discharge	“patient left before physical exam”	“Removed by security w/o being seen”
		“no diagnosis, patient was uncooperative, threatening, etc. left early”

## Interpretation

This is the first cohort study examining difficult behaviours in emergency departments among homeless, and low-income housed individuals. Difficult behaviours occurred in a quarter to a half of all emergency department encounters in these groups. Uncooperative behaviours made up three-quarters of difficult behaviours across all groups, while up to a fifth of difficult behaviours included violence. Nearly half of violent behaviours were severe, resulting in restraint or security interventions.

This study challenges conclusions that might be drawn from other studies suggesting that being homeless is an independant risk factor for difficult behaviours in the emergency department. [10,18,19,28,29] These studies considered emergency room records as the units of analysis in a single point in time. In contrast, this study followed individuals over time to examine their rates of presenting with difficult behaviours. This approach suggests that the vast majority of individuals in these populations do not exhibit difficult behaviours and when they do, it is because they are presenting intoxicated or otherwise acutely brain injured rather than being homeless or having a drinking problem. Rates of difficult behaviours were not found to be significantly different between the GH population and the LIH population, while the rates among the frequently intoxicated CHDP population were 14.3 times greater. Among the GH and LIH groups, 87% and 90%, respectively, were never involved in any recorded verbal abuse, while 90% and 95%, respectively, did not present with violent behaviours. In the CHDP group, more individuals exhibited difficult behaviours; however, they occurred in less than 10% of visits for 56% and 72% of subjects and less than half the time for the rest (Table 4). Out of 17 different measures examined, intoxication was a moderate to strong predictor of 11. Being identified as a problem drinker or being housed was significant for 1 or two. Membership in the chronically homeless problem drinking group was not a significant predictor of difficult behaviours when intoxication was taken into account. These types of variable interactions, suggest that the housing effect may be confounded by lower intoxicated presentation rates in the housed group. This analysis cannot resolve whether housing reduces substance use or whether reduced substance use makes it easier to be housed as the effects of changing housing status were not evaluated. Growing evidence that housing interventions lead to reductions in hospital utilization support the first interpretation.[30] Studies of difficult behaviours in emergency departments suggest that alcohol is implicated in 50% to 77% of abuse-of-staff incidents and 3% to 11% of incidents of leaving without being seen, however tests of association were not made in these studies. [18,20,31–33]

Multivariate analyses in this study suggest that being unconscious at some point during the visit is a strong predictor of being restrained, being verbally abusive, leaving against medical advice, and leaving before discharge. Unconsciousness was predictive of difficult behaviours independent of intoxication; this may suggest that both are the effects of an injured brain. This association with loss of consciousness was not found in the studies reviewed.[20,31–33] Emergency department resources may not be sufficient to meet the needs of many patients with severe intoxication or other injuries compounded by severe psychosocial problems and their resultant difficult behaviours.[34] Table 6 gives qualitative evidence suggestive of the how challenging and possibly inappropriate the current approach to care for these vulnerable populations can be for both the care providers and the patients. Difficult behaviours range from “refusing BP” to “setting bedsheets on fire” to being “put in 4 point restraint for staff safety”.

This study has several limitations. Difficult behaviours were likely undercounted to varying degrees due to recording bias. [20] Fernandes’ study of a comparable inner city hospital suggests a rate of 330 assaults in a year based on staff recall. [19] This study suggests 98. Over-recall in Fernandes’s study and under-recording in the current study may provide upper and lower bounds to the actual number. Other potential sources of undercount include exclusion of individuals from participation due to severe mental illness, severe intoxication, or not appearing as scheduled as well as including individuals in a shelter based harm reduction program that aimed to improve behaviours. The results of the current study may not be generalizable to other jurisdictions, subgroups, such as women and youth not included in this study, and present circumstances due to the age of the data. Older and more recent studies of difficult behaviours during this time suggestion that there has not been a change.[7–10] Some authors have suggested that difficult behaviours may increase as resources become more stretched.[34]

Intervention studies aimed at assisting those who are homeless especially those who are chronically homeless and heavy alcohol users should be a high priority. Approaches that reduce inappropriate and difficult emergency department visits, especially for those who are likely to present intoxicated, are needed. They are beginning to appear.[12,35,36] These studies however do not address or describe the impact of interventions on difficult behaviors. This study raises important topics for further research and program development, especially for vulnerable subgroups such as chronically homeless, alcohol-dependent men. These include the further evaluation and development of promising community and hospital-based interventions for those with severe problems related to heavy alcohol use, such as housing first, staff sensitivity and de-escalation skills training, compassionate care, harm reduction, and abstinence-based programs. These interventions may both reduce visits to the emergency department and rates of difficult behaviours.[22,30,35–37] Better understanding of staff, client, environmental, and care approach differences among emergency departments and within the community could also provide promising results.
